# *In vitro* anti-*Helicobacter pylori* activity of ilaprazole used alone and in combination with other components of quadruple therapy

**DOI:** 10.1128/spectrum.00593-25

**Published:** 2025-09-24

**Authors:** Zhimeng Zhang, Lu Yang, Wei Sun, Xinxin Hu, Tongying Nie, Lang Sun, Congran Li, Xinyi Yang, Xi Lu, Jing Pang, XueFu You

**Affiliations:** 1 Beijing Key Laboratory of Technology and Application for Anti-Infective New Drugs Research and Development, Institute of Medicinal Biotechnology, Chinese Academy of Medical Sciences and Peking Union Medical College 220735, Beijing, China; 2 Division for Medicinal Microorganism-Related Strains, CAMS Collection Center of Pathogenic Microorganisms, Chinese Academy of Medical Sciences & Peking Union Medical College 12501, Beijing, China; 3 Livzon Pharmaceutical Group Inc. 208329 , Zhuhai, China; 4 State Key Laboratory of Bioactive Substances and Functions of Natural Medicines, Institute of Medicinal Biotechnology, Peking Union Medical College and Chinese Academy of Medical Sciences 12501, Beijing, China; University of Georgia College of Veterinary Medicine, Athens, Georgia, USA

**Keywords:** ilaprazole, *H. pylori*, *in vitro *activity, synergy

## Abstract

Ilaprazole, a proton pump inhibitor, has been approved and marketed in Korea and China for the treatment of gastric ulcer, duodenal ulcer, gastroesophageal reflux disease, and erosive esophagitis. This study evaluated the *in vitro* antibacterial activity of ilaprazole against *Helicobacter pylori* (*H. pylori*), both as a single agent and in combination with other components used in the standard quadruple therapy. The antibacterial activity of ilaprazole was tested on 25 *H*. *pylori* strains, including the clinical isolates resistant to clarithromycin (CLA), amoxicillin (AMX), levofloxacin, and/or metronidazole. Antibacterial activities and killing kinetics were evaluated by the minimal inhibitory concentration (MIC) and time-kill curve determination, respectively. Synergistic effects were assessed in checkerboard and time-kill assays. Resistance development was assessed through serial passage over 12 cycles. Ilaprazole exhibited potent *in vitro* antibacterial activity against *H. pylori*, with MIC_50_ and MIC_90_ values of 8 µg/mL, demonstrating activity against drug-resistant strains. When combined with CLA, ilaprazole showed synergistic effects against 36% of the tested strains. Notably, the quadruple combination of ilaprazole + AMX + CLA + bismuth potassium citrate exhibited an obvious synergistic effect. Importantly, repeated exposure to ilaprazole over 12 passages did not induce resistance. These findings highlight the promising *in vitro* antibacterial activity of ilaprazole against *H. pylori*, including drug-resistant strains, and its potential to enhance the efficacy of quadruple therapy. The study supports the inclusion of ilaprazole in treatment regimens for *H. pylori* infections, offering a compelling rationale for its clinical use.

**IMPORTANCE:**

*H. pylori* infection remains a major global health issue, contributing to a wide range of gastric diseases. Despite current treatment regimens, rising antibiotic resistance limits their effectiveness, emphasizing the need for novel therapeutic approaches. This study highlights the promising *in vitro* antibacterial activity of ilaprazole against *H. pylori*, including drug-resistant strains. Ilaprazole not only exhibits direct antimicrobial effects but also enhances the efficacy of combination therapy, particularly in quadruple therapy regimens, which are recommended as first-line treatment. The findings demonstrate that ilaprazole, in combination with clarithromycin, shows synergistic effects, offering a potential solution to overcome antibiotic resistance challenges. Importantly, repeated exposure to ilaprazole did not induce resistance, a critical factor for its long-term use. These results provide compelling evidence for ilaprazole’s inclusion in clinical treatment strategies, contributing to improved eradication rates and better patient outcomes in *H. pylori* management.

## INTRODUCTION

*Helicobacter pylori* (*H. pylori*), a gram-negative microaerophilic pathogen, is recognized as a primary etiological agent in the development of chronic gastritis, peptic ulcer disease, gastric mucosa-associated lymphoid tissue lymphoma, and gastric cancer (1). It infects roughly half of the global population, particularly in developing countries, leading to significant health burdens (1). Eradication of *H. pylori* has been demonstrated to facilitate the healing of peptic ulcers and significantly reduce the incidence of gastric cancer (2). Although standard treatment regimens, which typically include proton pump inhibitors (PPIs) combined with two antibiotics such as clarithromycin (CLA), amoxicillin (AMX), levofloxacin (LEV), or metronidazole (MTZ), have been effective, rising rates of antimicrobial resistance (particularly to clarithromycin and metronidazole) are compromising eradication success (3). In response, current clinical guidelines, including the Maastricht VI Consensus, recommend a 14-day course of bismuth-based quadruple therapy as the preferred first-line treatment for *H. pylori* infection (4).

PPIs suppress gastric acid production by inhibiting H^+^/K^+^ ATPase, an enzyme localized to the canalicular membrane of gastric parietal cells and responsible for the secretion of hydrochloric acid, and were traditionally used for gastric symptom relief (5, 6). For the *H. pylori* eradication therapy, the inhibition of gastric acid by PPI enables *H. pylori* to enter an active replication state and show higher sensitivity to antibiotics (7). Besides, antibiotics exhibit higher stability to conduct better eradication in a neutral environment (7). Previous studies suggest that PPIs may also exhibit direct antibacterial activity against *H. pylori*, including strains resistant to conventional antibiotics (8–10). This multi-action mechanism, gastric acid suppression and antibacterial activity, rationalizes the application of PPIs in addressing current treatment challenges.

Ilaprazole, a novel PPI developed by Il Yang Pharmaceutical Co., Ltd. and Livzon Pharmaceutical Group Co., Ltd., has been approved and marketed in countries such as South Korea and China for the treatment of gastric ulcer, duodenal ulcer, gastroesophageal reflux disease (GERD), and erosive esophagitis. It demonstrates comparable tolerability, safety, and efficacy to omeprazole in the treatment of gastroduodenal ulcers at significantly lower doses than most clinically used PPIs (11, 12). Among the newer generation of PPIs, ilaprazole stands out due to its lower potential for drug interactions (13), a longer plasma half-life, sustained acid suppression, and a favorable efficacy-safety profile (14). Ilaprazole undergoes primary metabolism via CYP3A4 with minor CYP2C19 involvement (12, 15). This metabolic profile is clinically significant, given that CYP2C19 shows genetic polymorphism, leading to substantial inter-individual pharmacokinetic variability, especially among Asian populations (16). Unlike CYP2C19-dependent PPIs, ilaprazole demonstrates more consistent pharmacokinetics across genotypes, enhancing therapeutic reliability and minimizing genotype-driven adverse effects. Despite its established role in managing acid-related disorders, its utility in *H. pylori* eradication regimens has not been fully investigated. Particularly, whether ilaprazole possesses intrinsic antibacterial activity against *H. pylori*, especially in the context of increasing antibiotic resistance, remains unclear nor does its potential contribution to *H. pylori* eradication therapy. Furthermore, the direct synergistic anti-*H*. *pylori* effects *in vitro* of ilaprazole in combination with other components of quadruple therapy were seldom investigated.

Given the urgent need for novel strategies to combat *H. pylori* infections, this study aims to assess the anti-*H*. *pylori* activities of ilaprazole, taking other frequently used PPIs (omeprazole, esomeprazole, and vonoprazan) as comparators. It further seeks to elucidate the role of ilaprazole in enhancing the antibacterial efficacy of combination therapy against resistant strains, providing insights into optimizing clinical strategies for managing *H. pylori* infections.

## MATERIALS AND METHODS

### Antimicrobial susceptibility testing

The *H. pylori* strains utilized in this investigation were sourced from the Collection Center of Pathogen Microorganism of Chinese Academy of Medical Sciences (CAMS-CCPM-A) in China, comprising 2 reference strains and 23 clinical isolates, with 21 strains obtained from a Shanghai-based hospital between 2022 and 2023. Ilaprazole was supplied by Livzon Pharmaceutical Group Inc. (China). The MICs were assessed using the agar dilution technique in accordance with the Clinical and Laboratory Standards Institute (CLSI) M45 guidelines (15). Briefly, 100 µL aliquots of frozen stocks were inoculated onto Columbia agar supplemented with 5% defibrinated sheep blood and incubated under microaerobic conditions (10% CO_2_) at 37°C for 72 h. Subsequently, bacterial suspensions were adjusted to a 2.0 McFarland turbidity (approximately 10^8^ CFU/mL) and subcultured for an additional 48 h with shaking. The second-passage cultures were standardized to 2.0 McFarland, and aliquots of 1 µL were then inoculated onto Mueller-Hinton (MH) agar plates containing 5% defibrinated sheep blood and serially diluted test compounds. All compounds were prepared in two-fold serial dilutions: amoxicillin from 0.001 to 1  µg/mL and all other compounds from 0.03 to 128  µg/mL. Plates were incubated at 37°C for 72 h. *H. pylori* ATCC 43504 served as the quality control strain. MICs were defined as the lowest drug concentrations that inhibited visible bacterial growth. The experiments were performed twice on different days. For a certain drug, if the MIC values recorded were identical, the values were regarded as the MICs. If not, a third test would be performed, and the value recorded twice in the three tests is regarded as the MIC.

### Checkerboard assay for synergy study

The synergistic effects of drug combinations were evaluated using the agar dilution checkerboard method. MH blood agar plates (containing 5% defibrinated sheep blood) were prepared with varying concentrations of drug A and drug B. The drug combinations and their concentrations tested are as follows: ilaprazole at 0.25–16 µg/mL and CLA at 0.004–64 µg/mL; esomeprazole at 2–64 µg/mL and CLA at 0.004–64 µg/mL; ilaprazole at 0.25–16 µg/mL and AMX at 0.00025–0.5 µg/mL; esomeprazole at 2–64 µg/mL and AMX at 0.00025–0.5 µg/mL; ilaprazole at 0.25–16 µg/mL and bismuth potassium citrate at 0.25–16 µg/mL; esomeprazole at 2–64 µg/mL and bismuth potassium citrate at 0.25–16 µg/mL; CLA at 0.004–64 µg/mL and bismuth potassium citrate at 0.25–16 µg/mL; AMX at 0.00025–0.5 µg/mL and bismuth potassium citrate at 0.25–16 µg/mL. Bacterial suspensions adjusted to 2.0 McFarland were spot inoculated (1 µL) onto the plates. Following incubation at 37°C for 72 h under microaerobic conditions, the fractional inhibitory concentration index (FICI) was calculated as follows: FICI = (MIC of drug A in combination/MIC of drug A alone) + (MIC of drug B in combination/MIC of drug B alone). Synergy was defined as an FICI ≤0.5, additive effects as 0.5 < FICI ≤ 4, and antagonism as FICI >4.

### Time-kill assays

Time-kill experiments were performed to assess the bactericidal dynamics of ilaprazole, both alone and in combination, following CLSI guidelines (17). Two susceptible *H. pylori* strains (ATCC 43504 and CCPM(A)-P-372329) and two resistant strains (SS1 and CCPM(A)-P-3722159) were included in the study. The frozen stocks of 100 µL were inoculated onto Columbia agar supplemented with 5% defibrinated sheep blood and incubated anaerobically at 37°C for 72 h. Then, the collected bacteria suspending and adjusting to 2.0 McFarland were diluted 100-fold with brain heart infusion (BHI) broth supplemented with 10% (vol/vol) fetal bovine serum (FBS) and incubated anaerobically at 37°C with shaking for 24–48 h until reaching exponential phase of growth. Cultures were diluted to approximately 2 × 10^6^ CFU/mL, and drugs were added individually or in combination at specified concentrations. Cultures were incubated at 37°C with shaking, and colony counts were determined at 0, 4, 8, 24, 48, and 72 h by plating onto Columbia blood agar containing 5% defibrinated sheep blood. Growth controls without antimicrobial agents were included for each strain. Time-kill assays were performed twice independently, and for each time point from a single experiment, the mean values with standard deviations of three technical replicates were plotted. The limit of detection of the assay was 100 CFU/mL. Synergy was identified as a ≥2 log_10_ reduction in colony counts for the combination compared to the most active single agent at the corresponding time point (18).

### Resistance development by serial passage

The potential for resistance induction was evaluated by serially passaging *H. pylori* ATCC 43504 in sub-MIC concentrations of antimicrobial agents over 12 passages (36 days). Ilaprazole was used at 1 µg/mL, while amoxicillin, clarithromycin and esomeprazole were included as controls at a concentration of 0.0075, 0.0075 and 8 µg/mL, under identical conditions. Bacterial suspensions were adjusted to 2.0 McFarland and diluted 20-fold in BHI broth supplemented with 10% (vol/vol) FBS and antimicrobial agents at 1/4 MIC. Cultures were incubated at 37°C with shaking under microaerobic conditions for 72 h per passage. MICs were determined for each passage alongside the non-induced parental strain to monitor resistance development.

### Membrane potential measurement

Membrane potential measurements were performed as described previously (19, 20). In brief, *H. pylori* cells (OD_600_ = 0.4) were collected and washed with a buffer solution consists of phosphate-buffered saline (PBS), 100 mM KCl, 20 mM glucose. The resuspended cells were loaded with 5.0 µM potentiometric fluorophore 3,3′-dipropylthiadicarbocyanine iodide, DiSC_3_(5), dissolved in DMSO, and incubated in dark for 30 min. After the addition of the test compound, the fluorescence was recorded (excitation 610 nm and emission 660 nm) every 5 min. Valinomycin and carbonyl cyanide m-chlorophenyl hydrazone (CCCP) were used as controls.

## RESULTS

### Antibacterial activity of ilaprazole against *H. pylori* strains

The MICs of the tested compounds, including frequently used PPIs, antibiotics, and bismuth in quadruple therapy, against 25 *H*. *pylori* strains are summarized in Table 1. Among the 25 *H*. *pylori* strains used in this experiment, 3 isolates were resistant to amoxicillin, 9 isolates were resistant to clarithromycin, 8 isolates were resistant to metronidazole, and 7 isolates were resistant to levofloxacin. The results indicate that, beyond its primary function of reducing gastric acid secretion, ilaprazole exhibits favorable *in vitro* antibacterial activity against *H. pylori* with MICs ranging from 2 to 8 µg/mL. The MIC_50_ and MIC_90_ values for the 25 tested *H. pylori* strains were both 8 µg/mL. The anti-*H*. *pylori* activity of ilaprazole was greater than that of omeprazole and esomeprazole, for which MIC_50_ and MIC_90_ values were 32 µg/mL. For the drug-resistant strains, ilaprazole demonstrated comparable antibacterial activity with MIC values similar to those of the susceptible strains. While vonoprazan did not exhibit anti-*H*. *pylori* activity at concentrations up to 128 µg/mL. Bismuth potassium citrate also demonstrated excellent *in vitro* antibacterial effects against *H. pylori* with MICs ranging from 2 to 8 µg/mL. The findings from this study demonstrate that ilaprazole possesses significant *in vitro* antibacterial activity against *H. pylori*, surpassing the activity of esomeprazole, omeprazole, and vonoprazan.

**TABLE 1:** MICs of the tested proton pump inhibitors, antibiotics, and bismuth against standard and clinical isolated *H. pylori* strains^*e*^ (MIC: µg/mL)

Strain	Ilaprazole	Esomeprazole	Omeprazole	Vonoprazan	Bismuth potassium citrate	CLA^*a*^	MTZ^*b*^	LEV^*c*^	AMX^*d*^
CCPM(A)-P-372302	4	16	32	>128	4	0.06	1	0.5	0.015
CCPM(A)-P-372303	4	16	32	>128	4	32	64	16	0.06
CCPM(A)-P-372304	4	16	32	>128	4	16	1	8	0.03
CCPM(A)-P-372315	8	32	32	>128	4	16	1	8	0.25
CCPM(A)-P-372316	4	16	16	>128	4	16	1	0.5	0.015
CCPM(A)-P-372317	8	32	32	>128	4	32	32	16	0.008
CCPM(A)-P-372320	2	16	16	>128	2	≤0.03	1	0.25	0.002
CCPM(A)-P-372321	8	32	32	>128	8	0.06	2	0.5	0.03
CCPM(A)-P-372322	8	32	32	>128	4	0.06	64	0.25	0.06
CCPM(A)-P-372323	8	32	32	>128	4	0.06	64	8	0.06
CCPM(A)-P-372324	4	32	32	>128	4	≤0.03	64	0.25	0.004
CCPM(A)-P-372325	4	32	32	>128	4	0.06	2	0.25	0.015
CCPM(A)-P-372326	8	32	32	>128	4	0.06	4	0.25	0.002
CCPM(A)-P-372327	8	32	32	>128	4	≤0.03	2	0.25	0.008
CCPM(A)-P-372329	8	32	32	>128	4	≤0.03	64	0.25	0.125
CCPM(A)-P-372330	4	16	16	>128	4	≤0.03	64	0.5	0.015
CCPM(A)-P-372331	8	32	32	>128	8	0.06	2	0.25	0.008
CCPM(A)-P-372332	4	16	16	>128	4	8	1	4	0.004
CCPM(A)-P-372334	8	32	32	>128	8	16	1	0.25	0.125
CCPM(A)-P-372335	8	32	32	>128	4	≤0.03	2	0.25	0.008
CCPM(A)-P-372336	8	32	32	>128	4	16	2	0.25	0.03
ATCC 700392	8	32	32	>128	8	≤0.03	2	0.25	0.03
SS1	4	32	32	>128	2	≤0.03	0.25	0.25	0.25
CCPM(A)-P-3722159	4	32	16	>128	4	16	2	16	0.25
ATCC 43504	8	32	32	>128	8	≤0.03	64	0.5	0.03

^*a*^ CLA: MIC breakpoints against CLA for *H. pylori*: ≤0.25 µg/mL for susceptible and >0.25 µg/mL for resistant, according to EUCAST.

^*b*^ MTZ: MIC breakpoints against MTZ for *H. pylori*: ≤8 µg/mL for susceptible and >8 µg/mL for resistant, according to EUCAST.

^*c*^ LEV: MIC breakpoints against LEV for *H. pylori*: ≤1 µg/mL for susceptible and >1 µg/mL for resistant, according to EUCAST.

^*d*^ AMX: MIC breakpoints against AMX for *H. pylori*: ≤0.125 µg/mL for susceptible and >0.125 µg/mL for resistant, according to EUCAST.

^*e*^ Quality control range for antibiotics against *H. pylori* ATCC 43504 provided by CLSI (agar dilution method): AMX, 0.015–0.12 µg/mL; CLA, 0.015–0.12 µg/mL; MTZ, 64–256 µg/mL.

**Fig 1:**
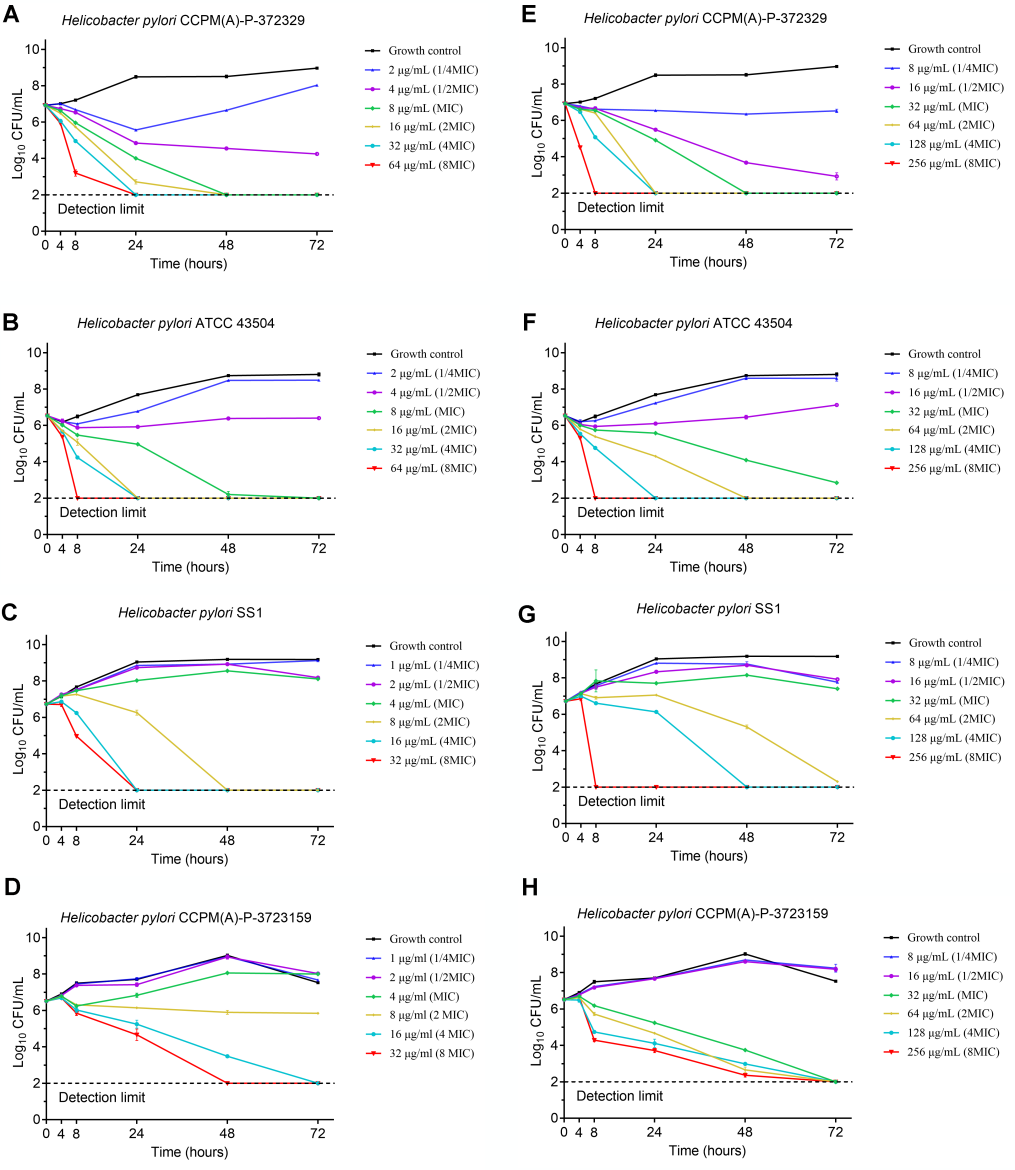
Time-kill curves of the *H. pylori* strains exposed to 1/4, 1/2, 1, 2, 4 and 8 MICs of ilaprazole and esomeprazole, respectively (mean ± SD, *n* = 3). (**A–D**) Time-kill curves of ilaprazole against *H. pylori* CCPM(A)-P-372329, ATCC 43504, SS1, and CCPM(A)-P-3722159. (**E–H**) Time-kill curves of esomeprazole against *H. pylori* CCPM(A)-P-372329, ATCC 43504, SS1, and CCPM(A)-P-3722159. The numbers of viable bacteria in the broth after each exposure period were enumerated, and the results were expressed as CFU/mL.

The time-kill results are shown in Fig. 1. Esomeprazole, the S-isomer of omeprazole and the most widely used PPI in clinical treatment for *H. pylori* eradication, was set as control. The strains selected for the time-kill study included a reference strain (ATCC 43504), a commonly used *in vivo* model strain with high gastric colonization capacity (SS1), and two clinical isolates of CCPM(A)-P-372329 (susceptible to AMX and CLA) and CCPM(A)-P-3722159 (resistant to AMX and CLA). This strategically diverse selection enables rigorous assessment of ilaprazole’s antibacterial dynamics across strains exhibiting differential antibiotic susceptibility profiles and biological relevance, thereby ensuring comprehensive evaluation of its antimicrobial activity. Ilaprazole and esomeprazole exhibited concentration-dependent bactericidal effects against the four tested *H. pylori* strains. The results indicated that ilaprazole was less effective against the resistant strains compared to the susceptible ones at the same concentrations. At the same concentration, ilaprazole demonstrated significantly better antibacterial activity compared to esomeprazole. For all strains tested, significant bactericidal effects of ilaprazole and esomeprazole were noted at 48 and 72 h post-inoculation at the concentration of 4 and 8 MICs.

### Synergistic effect of ilaprazole against *H. pylori in vitro*

The results of the checkerboard assay were shown in Tables 2 to 5. Ilaprazole and esomeprazole, when used in combination with clarithromycin, both exhibited a certain degree of synergistic effect. The combination of ilaprazole and CLA showed synergistic effects against 36% of the *H. pylori* strains (9 out of 25 strains) with the FIC indices ranging from 0.1875 to 0.5 and a median FIC index of 0.3917. The combination of esomeprazole and CLA also showed synergistic effects against 40% of the *H. pylori* strains (10 out of 25 strains) with the FIC indices ranging from 0.2813 to 0.5 and a median FIC index of 0.375. However, as shown in Tables 3 and 4, the combination of ilaprazole or esomeprazole with AMX and bismuth potassium citrate did not show significant synergistic effects against *H. pylori*, indicating that the benefits of combination therapy may be specific to certain antibiotics. As shown in Table 5, the combination of AMX and bismuth potassium citrate did not show synergistic effects against *H. pylori*, and the combination of CLA and bismuth potassium citrate only exhibited synergy on a small proportion of strains (4/25). In addition, there is no synergy between AMX and CLA as reported (21). In summary, only the combination of ilaprazole and CLA demonstrated significant synergy among all the pairwise combinations.

**TABLE 2:** Effects of ilaprazole and esomeprazole in combination with CLA against *H. pylori* strains in checkerboard assay^*a*^

Strain	Ilaprazole combined with CLA						Esomeprazole combined with CLA					
	MIC (µg/mL) alone		MIC (µg/mL) combined		FICI	Synergistic effect	MIC (µg/mL) alone		MIC (µg/mL) combined		FICI	Synergistic effect
	CLA	Ilaprazole	CLA	Ilaprazole			CLA	Esomeprazole	CLA	Esomeprazole		
CCPM(A)-P-372302	0.015	8	0.004	4	0.7667	−^*b*^	0.015	16	0.008	8	1.0333	−
CCPM(A)-P-372303	16	8	2	4	0.625	−	16	16	2	4	0.375	+^*c*^
CCPM(A)-P-372304	32	4	8	1	0.5	+	32	32	4	8	0.375	+
CCPM(A)-P-372315	16	8	2	2	0.375	+	16	32	0.5	8	0.2813	+
CCPM(A)-P-372316	16	8	2	2	0.375	+	8	16	2	4	0.5	+
CCPM(A)-P-372317	16	8	2	4	0.625	−	8	16	8	2	1.125	−
CCPM(A)-P-372320	0.06	8	0.008	4	0.6333	−	0.03	16	0.015	8	1	−
CCPM(A)-P-372321	0.125	8	0.008	2	0.314	+	0.03	16	0.008	8	0.7667	−
CCPM(A)-P-372322	0.06	8	0.015	4	0.75	−	0.06	16	0.015	8	0.75	−
CCPM(A)-P-372323	0.06	8	0.008	4	0.6333	−	0.03	16	0.008	8	0.7667	−
CCPM(A)-P-372324	0.015	8	0.008	0.5	0.5958	−	0.015	16	0.004	4	0.5	+
CCPM(A)-P-372325	0.06	8	0.03	2	0.75	−	0.06	16	0.03	8	1	−
CCPM(A)-P-372326	0.06	8	0.03	2	0.75	−	0.06	32	0.004	16	0.5667	−
CCPM(A)-P-372327	0.06	8	0.03	2	0.75	−	0.125	16	0.03	8	0.74	−
CCPM(A)-P-372329	0.03	8	0.008	2	0.5	+	0.06	16	0.008	8	0.6333	−
CCPM(A)-P-372330	0.03	4	0.015	2	1	−	0.03	16	0.008	8	0.7667	−
CCPM(A)-P-372331	0.125	8	0.03	2	0.49	+	0.06	16	0.03	8	1	−
CCPM(A)-P-372332	16	4	4	2	0.75	−	16	16	8	4	0.75	−
CCPM(A)-P-372334	32	8	4	0.5	0.1875	+	32	32	4	8	0.375	+
CCPM(A)-P-372335	0.015	8	0.004	4	0.7667	−	0.03	16	0.008	8	0.7667	−
CCPM(A)-P-372336	16	8	8	1	0.625	−	32	32	4	8	0.375	+
ATCC 43504	0.03	4	0.004	2	0.6333	−	0.03	16	0.004	4	0.3833	+
ATCC 700392	0.03	4	0.004	2	0.6333	−	0.03	32	0.004	8	0.3833	+
SS1	0.03	8	0.008	1	0.3917	+	0.03	32	0.004	16	0.6333	−
CCPM(A)-P-3722159	32	8	8	2	0.5	+	32	32	8	8	0.5	+

^*a*^ An FICI value of ≤0.5 indicated synergistic activity, 0.5 < FICI ≤ 4 indicated indifferent activity, and FICI >4 indicated antagonistic activity.

^*b*^ “−” indicates synergistic effect was not observed.

^*c*^ “+” indicates synergistic effect was observed.

**TABLE 3:** Effects of ilaprazole and esomeprazole in combination with AMX against *H. pylori* strains in checkerboard assay

Strain	Ilaprazole combined with AMX						Esomeprazole combined with AMX					
	MIC (µg/mL) alone		MIC (µg/mL) combined		FICI	Synergistic effect	MIC (µg/mL) alone		MIC (µg/mL) combined		FICI	Synergistic effect
	AMX	Ilaprazole	AMX	Ilaprazole			AMX	Esomeprazole	AMX	Esomeprazole		
CCPM(A)-P-372302	0.008	4	0.00025	4	1.0313	−^*a*^	0.008	8	0.00025	8	1.0313	−
CCPM(A)-P-372303	0.03	4	0.00025	4	1.0083	−	0.03	8	0.00025	8	1.0083	−
CCPM(A)-P-372304	0.015	8	0.002	4	0.6333	−	0.015	16	0.00025	16	1.0167	−
CCPM(A)-P-372315	0.125	8	0.015	4	0.62	−	0.5	16	0.00025	16	1.0005	−
CCPM(A)-P-372316	0.008	4	0.001	2	0.625	−	0.008	16	0.004	4	0.75	−
CCPM(A)-P-372317	0.008	2	0.00025	2	1.0313	−	0.004	8	0.00025	8	1.0625	−
CCPM(A)-P-372320	0.004	4	0.001	2	0.75	−	0.002	16	0.001	8	1	−
CCPM(A)-P-372321	0.03	8	0.008	4	0.7667	−	0.06	32	0.015	8	0.5	+
CCPM(A)-P-372322	0.03	8	0.008	4	0.7667	−	0.06	16	0.00025	16	1.0042	−
CCPM(A)-P-372323	0.03	8	0.008	4	0.7667	−	0.03	16	0.015	8	1	−
CCPM(A)-P-372324	0.015	8	0.001	4	0.5556	−	0.008	16	0.004	8	1	−
CCPM(A)-P-372325	0.008	8	0.004	2	0.75	−	0.008	16	0.004	8	1	−
CCPM(A)-P-372326	0.004	4	0.002	2	1	−	0.002	16	0.00025	16	1.125	−
CCPM(A)-P-372327	0.015	8	0.001	4	0.5667	−	0.015	16	0.00025	16	1.0167	−
CCPM(A)-P-372329	0.06	8	0.03	4	1	−	0.06	16	0.00025	16	1.0042	−
CCPM(A)-P-372330	0.008	4	0.001	2	0.625	−	0.008	16	0.00025	8	0.5313	−
CCPM(A)-P-372331	0.008	8	0.001	4	0.625	−	0.008	16	0.004	4	0.75	−
CCPM(A)-P-372332	0.008	4	0.002	1	0.5	+^*b*^	0.008	16	0.001	8	0.625	−
CCPM(A)-P-372334	0.25	8	0.125	1	0.625	−	0.125	32	0.06	16	0.98	−
CCPM(A)-P-372335	0.008	8	0.002	4	0.75	−	0.008	32	0.004	8	0.75	−
CCPM(A)-P-372336	0.03	8	0.008	4	0.7667	−	0.03	16	0.015	8	1	−
ATCC 43504	0.015	4	0.00025	4	1.0167	−	0.015	16	0.00025	16	1.0167	−
ATCC 700392	0.03	4	0.00025	2	0.5083	−	0.03	16	0.015	8	1	−
SS1	0.25	8	0.125	4	1	−	0.25	16	0.00025	16	1.001	−
CCPM(A)-P-3722159	0.25	8	0.125	4	1	−	0.25	32	0.03	16	0.62	−

^*a*^ “−” indicates synergistic effect was not observed.

^*b*^ “+” indicates synergistic effect was observed.

**TABLE 4:** Effects of ilaprazole and esomeprazole in combination with bismuth potassium citrate against *H. pylori* strains in checkerboard assay

Strain	AMX combined with bismuth potassium citrate						CLA combined with bismuth potassium citrate					
	MIC (µg/mL) alone		MIC (µg/mL) combined		FICI	Synergistic effect	MIC (µg/mL) alone		MIC (µg/mL) combined		FICI	Synergistic effect
	Bismuth potassium citrate	AMX	Bismuth potassium citrate	AMX			Bismuth potassium citrate	CLA	Bismuth potassium citrate	CLA		
CCPM(A)-P-372302	4	0.008	2	0.002	0.75	−^*a*^	2	0.03	2	0.004	1.1333	−
CCPM(A)-P-372303	4	0.06	2	0.03	1	−	2	16	1	2	0.625	−
CCPM(A)-P-372304	4	0.015	4	0.00025	1.0167	−	4	8	0.25	2	0.3125	+^*b*^
CCPM(A)-P-372315	4	0.5	1	0.25	0.75	−	8	16	2	0.003	0.2502	+
CCPM(A)-P-372316	4	0.015	4	0.00025	1.0167	−	4	8	1	4	0.75	−
CCPM(A)-P-372317	4	0.004	4	0.00025	1.0625	−	2	16	1	2	0.625	−
CCPM(A)-P-372320	2	0.004	1	0.002	1	−	2	0.03	1	0.015	1	−
CCPM(A)-P-372321	8	0.03	4	0.015	1	−	8	0.06	8	0.004	1.0667	−
CCPM(A)-P-372322	8	0.06	4	0.03	1	−	8	0.03	1	0.015	0.625	−
CCPM(A)-P-372323	2	0.03	0.5	0.015	0.75	−	2	0.3	2	0.004	1.0133	−
CCPM(A)-P-372324	4	0.004	4	0.00025	1.0625	−	4	0.03	2	0.015	1	−
CCPM(A)-P-372325	4	0.008	4	0.00025	1.0313	−	4	0.06	2	0.015	0.75	−
CCPM(A)-P-372326	4	0.004	4	0.00025	1.0625	−	4	0.06	2	0.03	1	−
CCPM(A)-P-372327	8	0.008	4	0.00025	0.5313	−	4	0.06	2	0.015	0.75	−
CCPM(A)-P-372329	4	0.125	4	0.00025	1.002	−	4	0.125	2	0.06	1	−
CCPM(A)-P-372330	4	0.004	4	0.00025	1.0625	−	2	0.06	0.5	0.03	0.75	−
CCPM(A)-P-372331	8	0.004	4	0.00025	0.5625	−	8	0.06	8	0.004	1.0667	−
CCPM(A)-P-372332	4	0.004	4	0.00025	1.0625	−	4	8	1	2	0.5	+
CCPM(A)-P-372334	8	0.125	1	0.06	0.605	−	8	8	2	1	0.375	+
CCPM(A)-P-372335	4	0.004	2	0.002	1	−	4	0.015	2	0.004	0.7667	−
CCPM(A)-P-372336	4	0.03	2	0.008	0.7667	−	4	16	2	8	1	−
ATCC 43504	8	0.03	4	0.015	1	−	8	0.03	0.25	0.03	1.0313	−
ATCC 700392	8	0.03	4	0.015	1	−	8	0.06	4	0.015	0.75	−
SS1	4	0.25	4	0.00025	1.001	−	4	0.015	2	0.004	0.7667	−
CCPM(A)-P-3722159	8	0.25	4	0.125	1	−	8	8	4	2	0.75	−

^*a*^ “−” indicates synergistic effect was not observed.

^*b*^ “+” indicates synergistic effect was observed.

**TABLE 5:** Effects of AXM and CLA in combination with bismuth potassium citrate against *H. pylori* strains in checkerboard assay

Strain	AMX combined with bismuth potassium citrate						CLA combined with bismuth potassium citrate					
	MIC (µg/mL) alone		MIC (µg/mL) combined		FICI	Synergistic effect	MIC (µg/mL) alone		MIC (µg/mL) combined		FICI	Synergistic effect
	Bismuth potassium citrate	AMX	Bismuth potassium citrate	AMX			Bismuth potassium citrate	CLA	Bismuth potassium citrate	CLA		
CCPM(A)-P-372302	4	0.008	2	0.002	0.75	−^*a*^	2	0.03	2	0.004	1.1333	−
CCPM(A)-P-372303	4	0.06	2	0.03	1	−	2	16	1	2	0.625	−
CCPM(A)-P-372304	4	0.015	4	0.00025	1.0167	−	4	8	0.25	2	0.3125	+^*b*^
CCPM(A)-P-372315	4	0.5	1	0.25	0.75	−	8	16	2	0.003	0.2502	+
CCPM(A)-P-372316	4	0.015	4	0.00025	1.0167	−	4	8	1	4	0.75	−
CCPM(A)-P-372317	4	0.004	4	0.00025	1.0625	−	2	16	1	2	0.625	−
CCPM(A)-P-372320	2	0.004	1	0.002	1	−	2	0.03	1	0.015	1	−
CCPM(A)-P-372321	8	0.03	4	0.015	1	−	8	0.06	8	0.004	1.0667	−
CCPM(A)-P-372322	8	0.06	4	0.03	1	−	8	0.03	1	0.015	0.625	−
CCPM(A)-P-372323	2	0.03	0.5	0.015	0.75	−	2	0.3	2	0.004	1.0133	−
CCPM(A)-P-372324	4	0.004	4	0.00025	1.0625	−	4	0.03	2	0.015	1	−
CCPM(A)-P-372325	4	0.008	4	0.00025	1.0313	−	4	0.06	2	0.015	0.75	−
CCPM(A)-P-372326	4	0.004	4	0.00025	1.0625	−	4	0.06	2	0.03	1	−
CCPM(A)-P-372327	8	0.008	4	0.00025	0.5313	−	4	0.06	2	0.015	0.75	−
CCPM(A)-P-372329	4	0.125	4	0.00025	1.002	−	4	0.125	2	0.06	1	−
CCPM(A)-P-372330	4	0.004	4	0.00025	1.0625	−	2	0.06	0.5	0.03	0.75	−
CCPM(A)-P-372331	8	0.004	4	0.00025	0.5625	−	8	0.06	8	0.004	1.0667	−
CCPM(A)-P-372332	4	0.004	4	0.00025	1.0625	−	4	8	1	2	0.5	+
CCPM(A)-P-372334	8	0.125	1	0.06	0.605	−	8	8	2	1	0.375	+
CCPM(A)-P-372335	4	0.004	2	0.002	1	−	4	0.015	2	0.004	0.7667	−
CCPM(A)-P-372336	4	0.03	2	0.008	0.7667	−	4	16	2	8	1	−
ATCC 43504	8	0.03	4	0.015	1	−	8	0.03	0.25	0.03	1.0313	−
ATCC 700392	8	0.03	4	0.015	1	−	8	0.06	4	0.015	0.75	−
SS1	4	0.25	4	0.00025	1.001	−	4	0.015	2	0.004	0.7667	−
CCPM(A)-P-3722159	8	0.25	4	0.125	1	−	8	8	4	2	0.75	−

^*a*^ “−” indicates synergistic effect was not observed.

^*b*^ “+” indicates synergistic effect was observed.

**Fig 2:**
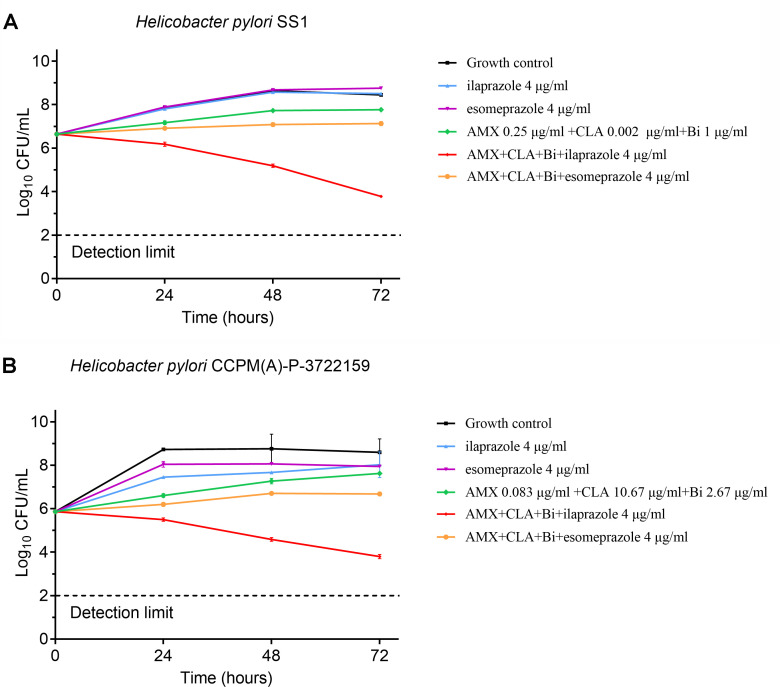
Synergistic effect of quadruple combinations *in vitro* (mean ± SD, *n* = 3). The numbers of viable bacteria in the broth after each exposure period were enumerated, and the results were expressed as CFU/mL. Each group is illustrated as follows: (**A**) *H. pylori* SS1 strain and (**B**) *H. pylori* CCPM(A)-P-3722159 strain.

The synergy was also observed in a time-kill assay for the combination of PPI and other typical components of the most classical quadruple therapy (AMX + CLA + bismuth potassium citrate) as illustrated in Fig. 2. When tested alone at sub-MIC concentrations, ilaprazole (4 µg/mL), esomeprazole (4 µg/mL), and the combination of AMX + CLA + bismuth potassium citrate (sub-MICs of each component) showed poor inhibitory potency and had no improvement in viable counts against both of the tested strains (SS1, an AMX-resistant strain, and CCPM(A)-P-3722159, a CLA-resistant strain) at 72 h compared with the growth control. Strikingly, when used in combination, a potent synergistic effect of quadruple combination of ilaprazole + AMX + CLA + bismuth potassium citrate was observed on both strains, leading to a significant reduction in viable counts by more than 2 log_10_ CFU/mL (4.73 and 4.22 log_10_ CFU/mL reduction for strains SS1 and CCPM(A)-P-3722159, respectively) compared to being used alone. A quadruple combination of esomeprazole + AMX + CLA + bismuth potassium citrate did not exhibit synergy in this concentration.

These findings offer evidence for the inclusion of ilaprazole in clinical quadruple therapy, enhancing its therapeutic rationale. By addressing both the symptomatic and microbial aspects of *H. pylori* infections, this research contributes to confirming the use of ilaprazole in the treatment strategies, potentially improving patient outcomes and combating antibiotic resistance in *H. pylori* management.

### Resistance induction of ilaprazole on *H. pylori*

**Fig 3:**
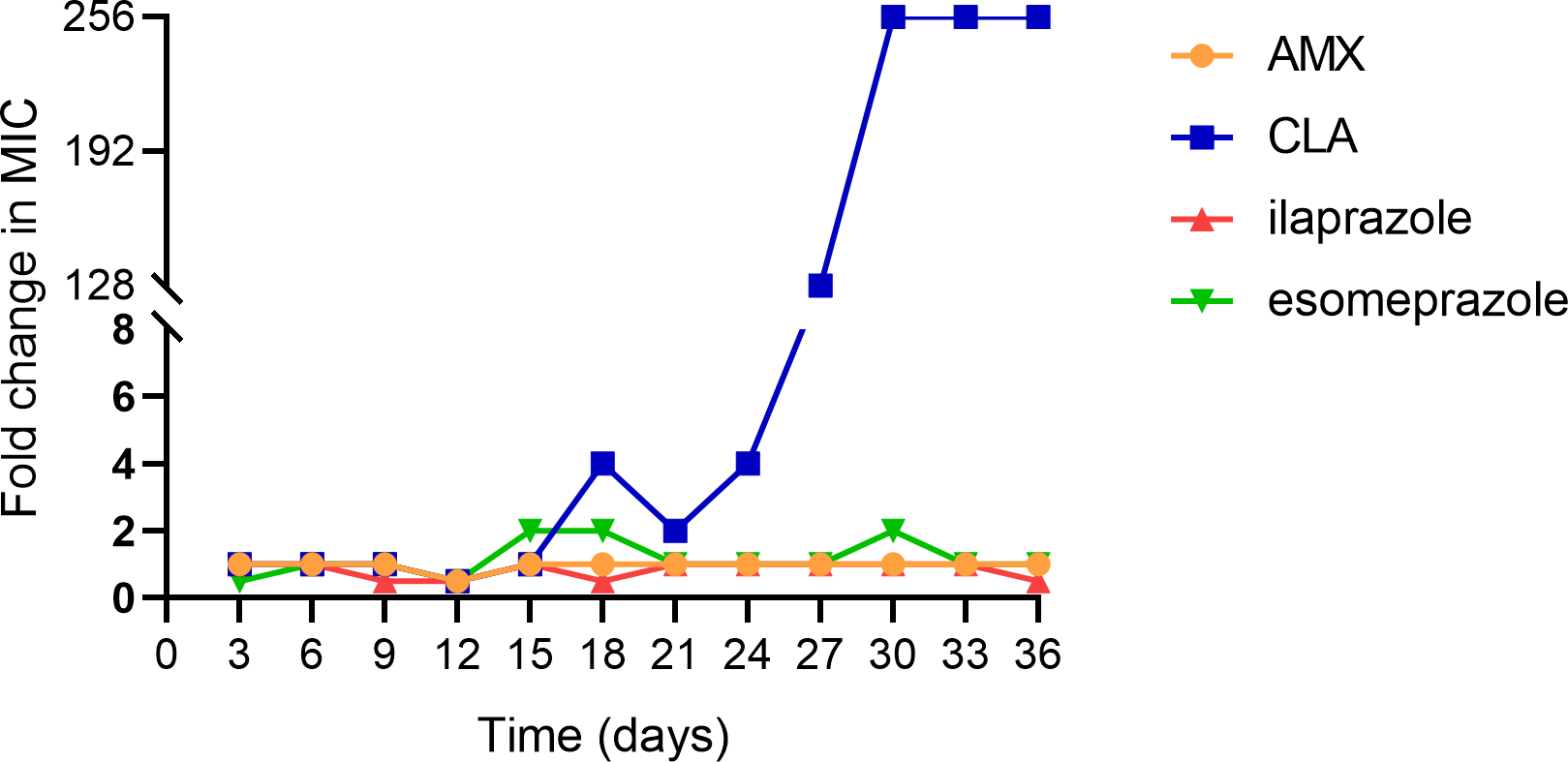
Broth dilution serial passage resistance induction studies. Serial passage resistance induction of ilaprazole was conducted with a concentration of 1 µg/mL (1/4 MIC) against *H. pylori* ATCC 43504, using 1/4 MIC of AMX, CLA, and esomeprazole as controls. MIC values for each agent were determined before and after each of 12 passages (1 passage per 3 days). Fold changes of MICs of each agent were calculated.

The development of bacterial resistance, often resulting from prolonged antibiotic exposure, is a major contributor to the failure of *H. pylori* eradication therapies. To assess the potential for resistance induction, ilaprazole and esomeprazole were evaluated using a 36-day serial passage assay with *H. pylori* ATCC 43504, a strain initially sensitive to clarithromycin and amoxicillin. As depicted in Fig. 3, continuous selective pressure from CLA over 12 passages led to the emergence of resistance, with the MIC increasing to and stabilizing at 4 µg/mL, representing a 256-fold rise compared to the baseline MIC. In contrast, repeated exposure to ilaprazole, esomeprazole, or AMX did not induce resistance in the tested *H. pylori* strain. These results indicate that ilaprazole and esomeprazole have a low propensity for resistance development, offering critical preclinical evidence to support their extended use in clinical applications.

### Ilaprazole role on *H. pylori* proton motive force

**Fig 4:**
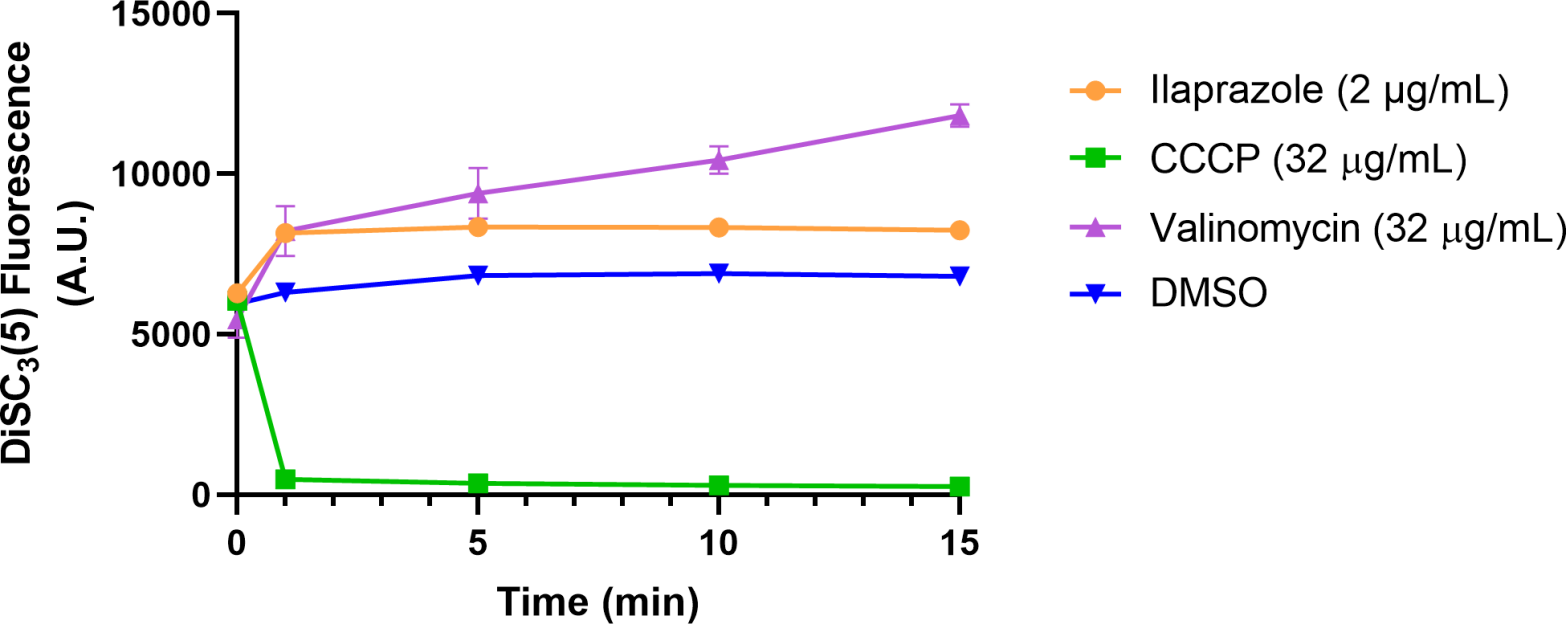
The effect of ilaprazole on membrane potential (mean ± SD, *n* = 3). Fluorescence probe DiSC_3_(5)-loaded *H. pylori* was treated with ilaprazole (2 µg/mL), valinomycin (32 µg/mL), and CCCP (32 µg/mL), and the fluorescence was recorded at different time points.

Proton motive force (PMF) sustains the electrochemical proton gradient across the bacterial inner membrane, driving ATP synthesis. PMF comprises two components of the electric potential (ΔΨ) and the transmembrane proton gradient (ΔpH). In this study, *H. pylori* cells were loaded with DiSC_3_(5), a potential-sensitive probe that accumulates in the cytoplasmic membrane in response to the electric potential and self-quenches its own fluorescence. Disruption of the electric potential triggers probe release into the extracellular milieu, increasing fluorescence. Conversely, dissipation of the transmembrane proton gradient induces compensatory elevation of the electric potential, enhancing DiSC_3_(5) uptake into the cytoplasmic membrane and, therefore, decreased fluorescence. Therefore, the dissipation of PMF can be determined by either increased DiSC_3_(5) fluorescence (ΔΨ loss and membrane depolarization) or decreased fluorescence (dissipation of transmembrane pH). CCCP, a protonophore disrupting PMF by decreasing transmembrane pH, and valinomycin, which can disrupt membrane potential, were used as positive controls. As shown in Fig. 4, ilaprazole and valinomycin treatment disrupted the membrane potential of *H. pylori*, as determined by increased fluorescence. In contrast, protonophore CCCP induced fluorescence decreasing by dissipating transmembrane pH.

## DISCUSSION

The published studies extensively cover ilaprazole as a proton pump inhibitor, focusing on its clinical efficacy for acid-related disorders (ulcers and GERD) and *H. pylori* eradication, detailed pharmacokinetics and pharmacodynamics, drug interaction profile, analytical methods for quantification, safety, and cost-effectiveness. Emerging research also explores its potential novel applications beyond acid suppression, including aspects on oncology and virology (22, 23). In this study, we evaluated the *in vitro* antibacterial activity of ilaprazole against *H. pylori* and its potential synergistic effects in combination with other components of quadruple therapy. Our findings demonstrate that ilaprazole exhibits antibacterial activity against both drug-sensitive and drug-resistant *H. pylori* strains, with MIC values comparable to or lower than those of conventional PPIs, such as omeprazole and esomeprazole. The observed antibacterial activity of ilaprazole reinforces the notion that certain PPIs possess direct antimicrobial properties beyond their established role in acid suppression. Bismuth potassium citrate also exhibits direct *in vitro* antibacterial effects against *H. pylori* in this study, which is also reported in other investigations previously (24, 25).

One of the key findings of this study is the synergy observed between ilaprazole and clarithromycin in 36% of tested strains. The checkerboard and time-kill assays confirmed that the combination of ilaprazole with clarithromycin resulted in enhanced bacterial inhibition compared to monotherapy. This suggests that ilaprazole may contribute to improved eradication rates when used as part of a quadruple regimen, particularly in cases where clarithromycin resistance is a concern. However, the combination of ilaprazole with amoxicillin or bismuth potassium citrate did not show significant synergy, indicating that the benefits of PPIs in combination therapy may be antibiotic specific.

Our study also highlights that ilaprazole, when included in a quadruple combination therapy (ilaprazole + AMX + CLA + bismuth potassium citrate), exhibited a potent synergistic effect in reducing bacterial counts. This observation is clinically relevant, as quadruple therapy is currently the recommended first-line treatment for *H. pylori* infections, particularly in regions with high antibiotic resistance. The enhanced efficacy of this combination suggests that replacing standard PPIs with ilaprazole in quadruple therapy could improve treatment outcomes.

Furthermore, resistance induction experiments showed that repeated exposure to ilaprazole did not lead to the development of resistance in *H. pylori*, in contrast to clarithromycin, which exhibited a marked increase in MIC over serial passages. This finding suggests that ilaprazole may have a lower potential for resistance development, supporting its utility in *H. pylori* eradication regimens.

In this study, synergy was observed between ilaprazole and clarithromycin, but not amoxicillin. The differential synergy may arise from the distinct mechanisms of the antibiotics and ilaprazole’s mode of action. Clarithromycin is a macrolide antibiotic that contains a 14-membered lactone ring bearing glycosidically linked amino sugar. As a protein synthesis inhibitor, clarithromycin must cross both the outer and inner membrane structure of bacteria to reach its ribosomal target. In contrast, amoxicillin, a β-lactam cell wall synthesis inhibitor, acts primarily in the periplasmic space without requiring inner membrane penetration. This mechanistic divergence suggests that ilaprazole may act on the inner membrane of *H. pylori*. Therefore, the effect of ilaprazole on membrane PMF was investigated in this study. Multiple antimicrobial agents are known to disrupt bacterial membranes by dissipating components of the PMF, specifically the electrical potential gradient or the proton gradient (26). This mechanism prompted our hypothesis that ilaprazole similarly compromises the PMF in *H. pylori*. The dissipation of *H. pylori* PMF was observed after the treatment of ilaprazole, as evidenced by increased DiSC_3_(5) fluorescence, which indicates membrane depolarization and the subsequent release of DiSC_3_(5) from the membrane. The synergy between ilaprazole and clarithromycin likely arises from ilaprazole-induced inner membrane potential disturbance, enhancing the accumulation of clarithromycin in cytoplasm. In contrast, the mechanism of amoxicillin is fundamentally less susceptible to potentiation by these specific ilaprazole-induced effects, explaining the lack of observed synergy. Furthermore, the absence of synergy between AMX and ilaprazole also suggests uninhibited bacterial protein synthesis may partially counteract ilaprazole’s activity, which requires further validation.

Despite the promising results, our study has certain limitations. First, the antibacterial activity of ilaprazole was evaluated *in vitro*, however, its efficacy in clinical settings remains unconfirmed. Second, while our study included multiple drug-resistant strains, further investigation involving a larger and more diverse collection of *H. pylori* isolates is needed to validate the generalizability of our findings. Finally, additional studies are warranted to elucidate the precise mechanism by which ilaprazole exerts its antibacterial effects against *H. pylori*.

### Conclusions

In conclusion, this study provides compelling evidence of ilaprazole’s *in vitro* antibacterial activity against *H. pylori*, including drug-resistant strains. The observed synergy with CLA and the potent effects of the quadruple combination highlight ilaprazole’s potential to enhance the efficacy of current treatment regimens.

These findings support the inclusion of ilaprazole in clinical quadruple therapy for *H. pylori* infections. By offering both acid suppression and direct antibacterial activity, ilaprazole addresses critical challenges in the treatment of *H. pylori*. Further clinical studies are warranted to confirm these results and establish optimized protocols for ilaprazole-based therapies.
